# Analysis of the role of mutations in the KMT2D histone lysine methyltransferase in bladder cancer

**DOI:** 10.1002/2211-5463.12600

**Published:** 2019-02-21

**Authors:** Beichen Ding, Libin Yan, Yucong Zhang, Zhize Wang, Yangjun Zhang, Ding Xia, Zhangqun Ye, Hua Xu

**Affiliations:** ^1^ Department of Urology Tongji Hospital Tongji Medical College Huazhong University of Science and Technology Wuhan China; ^2^ Institute of Urology of Hubei Province Wuhan China

**Keywords:** bladder cancer, copy number, histone lysine methyltransferase, KMT2D, mutations

## Abstract

Histone lysine methyltransferases (HMT) comprise a subclass of epigenetic regulators; dysregulation of these enzymes affects gene expression, which may lead to tumorigenesis. Here, we performed an integrated analysis of 50 HMTs in bladder cancer and found intrinsic links between copy number alterations, mutations, gene expression levels, and clinical outcomes. Through integrative analysis, we identified six HMT genes (*PRDM9*,*ASH1L*,*SETD3*,*SETD5*,*WHSC1L1*, and *KMT2D*) that may play a key role in the development and progression of bladder cancer. Of these six HMTs, histone lysine *N*‐methyltransferase 2D (*KMT2D*) exhibited the highest mutation rate in bladder cancer. Our comparison of the mRNA and miRNA expression profiles of mutated and wild‐type *KMT2D* suggested that two signaling pathways (FOX1–miR‐1224‐5p–DLK1 and HIF/GATA5–miR‐133a‐3p–DRD5) may mediate the tumor suppressive effect of the *KMT2D* mutation. In summary, our findings indicate that mutations in HMT genes, especially *KMT2D* mutation, may play a role in the development of bladder cancer.

**Abbreviations**
BCa: bladder cancer
CNA: copy number alteration
DEG: differentially expressed gene
FC: fold change
GSEA: Gene Set Enrichment Analysis
HMT: histone lysine methyltransferase
KMT2D: histone lysine *N*‐methyltransferase 2D
MTS: 3‐(4,5‐dimethylthiazol2‐yl)‐5‐(3‐carboxymethoxyphenyl)‐2‐(4‐sulfophenyl)‐2*H*‐tetrazolium, inner salt
TCGA: The Cancer Genome Atlas
TF: transcription factor

Bladder cancer (BCa) is a complex health problem worldwide with high incidence rate and mortality if not treated properly, and the morbidity of BCa ranks at fourth place in men and 11th place in women among all cancers 1. In the USA, 81 190 cases are expected to be diagnosed and the estimated deaths from BCa reached 17 240 in 2018 2. Two non‐overlapping subtypes (‘papillary’ and ‘non‐papillary’) have been identified according to different clinical phenotypes and genomic alterations, such as the activating mutations of *FGFR3* in papillary tumors and loss‐of‐function mutations in tumor suppressor genes *TP53* and *RB1* 3. Most muscle‐invasive BCas belong to the non‐papillary pathway and non‐muscle‐invasive BCas always represent the papillary subtype 4. New subtype‐specific treatment targets can be revealed by understanding the genetic and epigenetic alterations associated with different types of BCa.

Histone lysine methyltransferases (HMTs) and demethylases are subclasses of epigenetic regulators, which control the procedure of histone lysine methylation. Approximately 50 HMTs have been identified so far 5. According to structure, HMTs can be divided into two distinct functional enzymatic subtypes, SET (suppressor of variegation, enhancer of zeste, trithorax) domain‐containing methyltransferases and DOT1‐like lysine methyltransferases. Catalyzed by HMTs, lysines can be monomethylated (me1), dimethylated (me2) or trimethylated (me3), leading to diverse biological outcomes, such as chromatin compaction and gene expression 6.

In recent years, epigenetic changes in different diseases caused by HMTs were verified by an increasing number of studies. Dysregulation of HMTs leads to changes of downstream gene expression and results in the pathogenesis of various cancers, including BCa 7, 8. As a core subunit of polycomb repression complex 2, EZH2 catalyzes the trimethylation of K27 of H3, causing the repressing transcription of downstream genes. *EZH2* has been found to be overexpressed and is regarded as an oncogene in a wide range of human cancer types, such as BCa, breast cancer and renal cancer 8, 9, 10, 11. A growing number of studies have shown that genetic alterations in several biologically functional HMTs play an important role in the development and progression of cancer. Despite the emerging DNA and RNA sequencing data, such as The Cancer Genome Atlas (TCGA), there is still no systematic analysis of abnormities in genome and HMT expression among different subtypes of BCa. Besides, the relevance of genetic aberrations to clinical characteristics is still not completely clear. One of the histone H3 lysine 4‐methyltransferases, histone lysine *N*‐methyltransferase 2D (KMT2D, also known MLL2) was reported to have the highest mutation rate at 26.9% among all HMTs in BCa 12, and we further conducted a genomic consequence study of *KMT2D* mutations. Hence, this study aimed to perform an integrative genomic analysis of HMT, especially alterations of KMT2D and identify the functional consequences in the occurrence and progression of BCa.

## Materials and methods

### Samples with genomic and clinical data

A total of 426 bladder cancer (BLCA) samples including DNA copy number, gene expression, mutation, clinicopathological data and overall survival datasets were downloaded from TCGA at UCSC Xena and cBioPortal 13, 14. The copy number of HMTs was generated by the copy number algorithm GISTIC (Genomic Identification of Significant Targets in Cancer) algorithm analysis: ‘−2’ represents a homozygous deletion, ‘−1’ indicates a heterozygous deletion, ‘0’ represents diploid, ‘1’ indicates a low‐level gain, and ‘2’ signifies a high‐level amplification.

### Statistical analysis

Statistical analyses were performed using the r software (version 1.01; R Foundation for Statistical Computing, Vienna, Austria), prism (version 7.01; GraphPad Software Inc., La Jolla, CA, USA) and spss (version 18.0; SPSS Inc., Chicago, IL, USA). The correlations between copy number changes and mRNA expression of 50 HMTs in 426 sequenced BCa specimens were analyzed with Spearman and Pearson correlation tests. For RNA‐Seq and miRNA‐Seq data, we removed the genes/miRNAs which were not expressed and retained genes/miRNAs with counts per million (cpm) > 1 in more than one sample for further analysis. The edger package in r software was used for differential expression analysis of RNA‐Seq and miRNA. Significantly differentially expressed genes (DEGs) or differentially expressed miRNAs were defined by cutoffs: |log_2_(FC)| > 1 and *P* < 0.05. Heatmaps and volcano plots of gene/miRNA expression profiles in different subtypes of BCa were conducted by morpheus (https://software.broadinstitute.org/morpheus/) and r statistical software. Student's *t*‐test was used in calculating differences in mRNA expression levels of each HMT between papillary and non‐papillary BCa subtypes. The Kaplan–Meier survival curve was used to analysis the impact of copy number alteration or gene expression of different HMTs on survival. The potential target genes of miRNAs were identified by databases TarBase and miRTarBase 15, 16. Transcription factors (TFs) were annotated based on the JASPAR database 17.

### Gene function, pathway enrichment analysis and Gene Set Enrichment Analysis

Database for Annotation, Visualization, and Integrated Discovery (DAVID) v6.8 Beta (https://david-d.ncifcrf.gov/) and Gene Set Enrichment Analysis (GSEA) were applied for gene function and pathway enrichment analysis. The DAVID database was applied to investigate GO annotation and KEGG pathways of DEGs. GSEA was performed by gsea software (Version 2.2.2) from the Broad Institute (http://www.broad.mit.edu/gsea). An enrichment map was generated for visualization of the GSEA results. A normalized enrichment score and an adjusted *P* value were calculated to identify the hallmarks enriched in each phenotype.

### Establishment of *KMT2D* mutation‐specific co‐expression network

The correlation coefficient of any two DEGs was calculated based on the RNA‐Seq results, and pairs whose correlation coefficient was higher than 0.95 or lower than −0.95 were reserved in the network. A *KMT2D* mutation‐specific up‐regulated co‐expression network was built with 317 up‐regulated genes and 39 down‐regulated miRNAs whose targets were in up‐regulated genes. In a similar way, 203 down‐regulated genes and eight up‐regulated miRNAs whose targets are in down‐regulated genes formed the *KMT2D* mutation‐specific, down‐regulated network. Genes with 15 or more degrees in the down‐regulated network and 50 or more degrees in the up‐regulated network are shown in figures visualized by the cytoscape software 18.

### Cell culture, siRNA transfection

5637 and EJ1 cells were cultured in RPMI‐1640 (Thermo Fisher Scientific, Waltham, MA, USA) supplemented with 10% FBS (Gibco) in an atmosphere at 37 °C with 5% CO_2_.

In the RNA knockdown assay, cells grown in 12‐well plates were transfected with siRNAs with the GenMute™ transfection agent (SignaGen Laboratories, Rockville, MD, USA) according to manufacturer's protocols.

The *KMT2D* siRNA (5′‐GCTGCTATCGCTGTTCTATdTdT‐3′/5′‐ATAGAACAGCGATAGC AGCdTdT‐3′) and control siRNA were purchased from RiboBio Co., Guangzhou, China.

### Wound healing assays, cell proliferation, cell migration and invasion assays

siRNA‐transfected cells were plated into a six‐well plate at 90% confluence. A 2 mm injury line was made with a sterile scraper across the cell monolayer and cell migration was monitored at 0 and 24 h. Migration and invasion assays were performed using uncoated and Matrigel™‐coated Transwell^®^ inserts according to the manufacturer's instructions (CellTiter 96^®^ AQueous, Promega, Madison, WI, USA). Cell proliferation was estimated using the 3‐(4,5‐dimethylthiazol2‐yl)‐5‐(3‐carboxymethoxyphenyl)‐2‐(4‐sulfophenyl)‐2*H*‐tetrazolium, inner salt (MTS) method (Sigma‐Aldrich, St Louis, MO, USA) according to the manufacturer's instructions.

### Quantitative real‐time PCR

Total RNAs were extracted by MagZol (Invitrogen, Carlsbad, CA, USA) and cDNAs were synthetized by using SYBR Premix Ex TaqTM (TaKaRa, Kusatsu, Shiga, Japan). Real‐time PCR was performed by SYBR Green Realtime PCR Master Mix (TOYOBO, Kita‐ku, Osaka, Japan). Primers for the reaction are provided as followed: *DLK1* (forward: 5′‐CTGAAGGTGTCCATGAAAGAG‐3′, reverse: 5′‐GCTGAAGGTGGTCATGTCGAT‐3′), DRD5 (forward: 5′‐TCATCTATGCCTTCAACGCCGACT‐3′, reverse: 5′‐AGCTGCGATTTCCTTGTGGAAGAC‐3′), *GAPDH* (forward: 5′‐GTCATCATCTCCGCCCCTTCTGC‐3′, reverse: 5′‐GATGCCTGCTTCACCACCTTCTTG‐3′).

## Results

### HMT genetic aberration in bladder cancer

Somatic mutation and copy number alteration (CNA) are significant causes of the imbalance between oncogenes and tumor suppressor genes in carcinogenesis and progression of human cancer 19, 20. To obtain comprehensive genomic characterization of HMTs in BCa, we first performed a systematical analysis of 426 BCa sample sequencing data from TCGA database.

We analyzed the altered copy number and mutations of 50 HMTs encoded by the human genome in BCa (Table S1). Intriguingly, eight HMTs (*SETDB1*,* WHSC1L1*,* SETD5*,* PRDM9*,* ASH1L*,* SETMAR*,* MECOM*, and *PRDM14*) were identified with a rate of high‐level amplification more than 4% and three (*SETDB1*,* WHSC1L1* and *SETD5*) exhibited the highest rate at around 10%. The homozygous deletion rate of *SETDB2* and *WHSC1L1* is more than 2% (6.95%, 2.23%) in BCa. Additionally, somatic mutations of 13 HMT genes (*KMT2D*,* KMT2C*,* KMT2A*,* ASH1L*,* SETD2*,* PRDM16*,* PRDM9*,* SUV420H1*,* WHSC1*,* PRDM2*,* DOT1L*,* KMT2E*, and *NSD1*) existed in more than 5% of BCa samples. Notably, the mutation rate of *KMT2D* was up to 28.4%, which was the highest among HMTs in BCa and consistent with a previous study 12.

Thus, *KMT2D* probably played important roles in the occurrence and progression of BCa, and this was investigated in the following section.

### Expression profiling and copy number alterations of HMTs in bladder cancer

Driver oncogenes can be predicted by correlation between gene expression and copy number 5. Thus, we analyzed the correlation between copy number and gene expression level of HMTs from 426 BCa samples. Pearson and Spearman tests were applied to evaluate the correlations. The correlation coefficient orders of the two tests were similar, and HMTs were listed by the order of Pearson correlation coefficient. Fourteen HMTs exhibited a strong relative linear relationship (*R* > 0.5, *P* < 0.05) between CNA and expression with both statistical methods. *SETDB1* and *WHSC1L1* had the strongest correlation in Pearson and Spearman analysis, which is consistent with our previous findings for the high‐level amplification rate (shown in Table 1).

**Table 1:** Associations between CNA and expression, and comparison of mRNA expression between papillary and non‐papillary bladder cancer subtypes. Genes were ranked based on the Pearson correlation coefficient. Differentially expressed HMTs in the two subtypes are highlighted in bold

Gene ID	CNA/mRNA correlation		Papillary/non‐papillary comparison
	Pearson	Spearman	*t* statistic
*SETDB1*	0.794	0.718	2.337
*WHSC1L1*	0.782	0.742	−0.583
*SETDB2*	0.748	0.618	0.362
*SETD5*	0.677	0.590	1.971
*SETD3*	0.652	0.605	**−3.860**
*SUV420H1*	0.638	0.550	1.313
*EHMT1*	0.625	0.605	−1.493
*ASH1L*	0.625	0.605	−0.571
*SMYD4*	0.623	0.572	−1.624
*SETD8*	0.604	0.572	0.567
*SETD1A*	0.579	0.543	2.463
*SETD4*	0.577	0.576	**4.045**
*SMYD5*	0.564	0.481	−0.111
*PRDM4*	0.561	0.482	1.055
*SETMAR*	0.560	0.503	**4.735**
*SUV39H2*	0.545	0.474	**−2.612**
*SETD2*	0.543	0.604	1.950
*NSD1*	0.508	0.473	−1.188
*PRDM2*	0.488	0.473	1.343
*EZH1*	0.464	0.435	1.111
*SETD1B*	0.457	0.417	1.673
*DOT1L*	0.422	0.342	**2.658**
*SUV420H2*	0.422	0.383	1.141
*SMYD2*	0.406	0.390	−0.487
*SETD6*	0.405	0.469	2.108
*PRDM15*	0.370	0.409	2.064
*WHSC1*	0.358	0.296	−1.297
*PRDM11*	0.323	0.331	**−3.224**
*KMT2B*	0.273	0.546	**2.640**
*KMT2D*	0.237	0338	**2.686**
*EZH2*	0.223	0.226	−0.434
*KMT2C*	0.197	0.373	0.088
*SUV39H1*	0.178	0.116	−1.614
*PRDM1*	0.168	0.158	−4.126
*PRDM9*	0.163	0.094	0.478
*PRDM13*	0.159	0.173	−1.775
*PRDM12*	0.158	0.146	0.384
*PRDM10*	0.126	0.132	2.482
*PRDM8*	0.125	0.070	**−3.623**
*MECOM*	0.122	0.103	**4.860**
*KMT2A*	0.111	0.372	0.509
*PRDM1*	0.096	0.128	**−4.126**
*SMYD3*	0.086	0.158	0.409
*PRDM5*	0.077	0.024	**−2.954**
*PRDM6*	0.044	−0.006	**−3.103**
*PRDM14*	0.012	−0.009	−2.187
*PRDM16*	−0.004	−0.021	**−4.236**
*SMYD1*	−0.028	0.010	−1.519

We obtained expression profiles of 50 HMTs between tumor samples and peri‐tumor tissues. As shown in Fig. S1, *SMYD2*,* EZH2*, and *SUV420H2* were up‐regulated and *SETD7*,* PRDM1*,* PRDM8*,* PRDM6*, and *PRDM5* were down‐regulated in tumor. Furthermore, we compared the expression level of different subtypes of BCa by Student's *t*‐test (shown in Table 1). Results showed that *SETD3*,* PRDM1*,* SUV39H2*,* PRDM5*,* PRDM6*,* PRDM11*, and *PRDM16* were significantly up‐regulated in non‐papillary BCa while *KMT2D*,* KMT2B*,* MECOM*,* DOT1L*,* SETMAR*, and *SETD4* were down‐regulated, compared with papillary BCa (*P* < 0.01).

### Copy number alternation and expression level of HMTs are associated with clinical characteristics of bladder cancer patient

**Figure 1:**
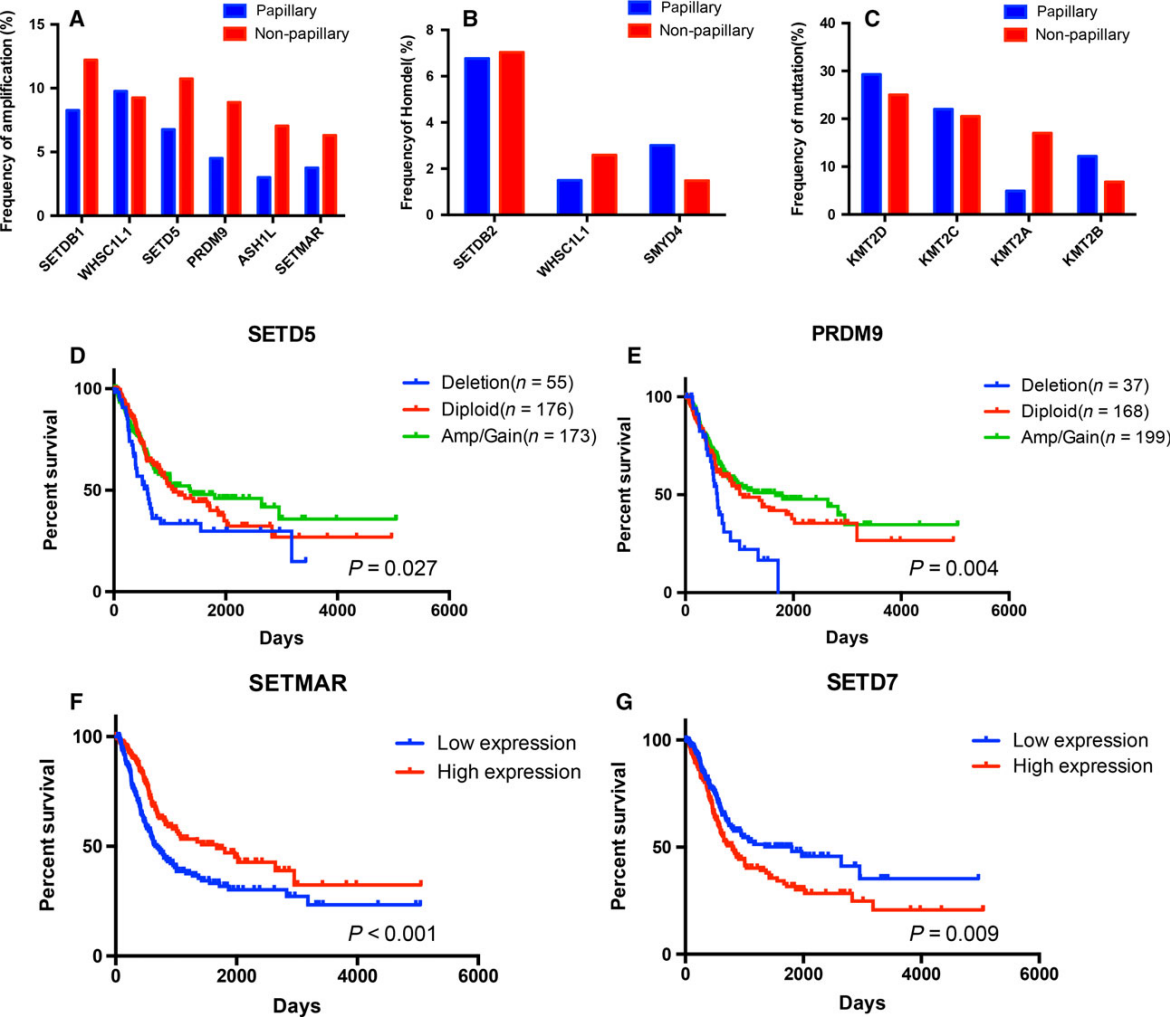
Copy number alterations and expression profiles of HMTs in different bladder cancer samples. (A–C) Amplification rate of six HMTs (A), homozygous deletion rate of three HMTs (B), and mutation rate of four HMTs (C) in 426 bladder cancer samples from TCGA between different subtypes of bladder cancer. (D–G) Kaplan–Meier plots of overall survival associated with copy number of *SETD5* (D) and *PRDM9* (E) and mRNA expression of *SETMAR* (F) and *SETD7* (G).

To determine whether HMT genomic alterations showed different pattern in different subtypes of BCa, we compared the mutation rate and CNA rate of HMTs between 133 papillary BCa samples and 270 non‐papillary BCa samples in TCGA database. With the exception of *WHSC1L1*, five (*SETDB1*,* SETD5*,* PRDM9*,* ASH1L*, and *SETMAR*) of the six top frequently amplified HMTs had obviously higher high‐level amplification rate in non‐papillary BCa (Fig. 1A). Of the three most homozygous deletion‐altered HMT genes, *WHSCIL1* exhibited higher alteration rate in non‐papillary while *SMYD4* exhibited the opposite (Fig. 1B). *KMT2D*,* KMT2C*, and *KMT2A* were most frequently mutated in papillary BCa, while the mutation rate of *KMT2A* was higher in non‐papillary BCa (Fig. 1C).

To explore the association between clinical outcomes and HMT genetic aberration in BCa, we investigated the association between CNA, mRNA expression, and overall patient survival in 426 samples. Samples were divided into three groups for each HMT: amp/gain (high‐level amplification/low‐level gain), diploid, and deletion (heterozygous and homozygous deletion). Survival analysis of among each HMT CNA subtype was performed. The results indicated copy number deletion of *PRDM9* and *SETD5* was significantly associated (*P* < 0.05) (Fig. 1D,E) with shorter survival. Also, we found that high *SETD7* and low *SETMAR* expression was correlated with shorter survival, by comparing low (*n* = 211) and high (*n* = 211) expression subgroups according to the mRNA expression of each HMT (Fig. 1F,G).

### Overall conclusion for identified HMTs in bladder cancer

Based on our previous results on analyzing genetic alternations and clinical outcome, Table 2 lists the integrative score of critical HMTs. Each category was marked as ‘+’ if an HMT met the criteria. As shown in Table 2, HMTs *PRDM9*,* ASH1L*,* SETD3*,* SETD5*,* WHSC1L1* and *KMT2D* had a score of more than 2, suggesting that these six HMTs may play critical roles in BCa oncogenesis.

**Table 2:** Integrative identification of critical HMTs in bladder cancer. CNA/mutations: amplification, deletion or mutation; CNA/mRNA correlation: associations between CNA and gene expression; expression: altered expression in RCC; survival: mRNA/CNA/mutations associated with patient survival

Gene	CNA/mutations	CNA/mRNA correlation	Expression	Survival	Total score
*PRDM9*	++		+	+	4
*ASH1L*	++	+			3
*SETD3*	+	+		+	3
*SETD5*	+	+		+	3
*WHSC1L1*	++	+			3
*KMT2D*	++			+	3
*KMT2C*	++				2
*MECOM*	++				2
*PRDM1*	+		+		2
*PRDM6*	+		+		2
*SETDB1*	+	+			2
*SETDB2*	+	+			2
*SETMAR*	+			+	2
*SUV420H2*	+		+		2
*SETD7*			+	+	2
*KMT2B*	+			+	2

### *KMT2D* mutations in bladder cancer

**Figure 2:**
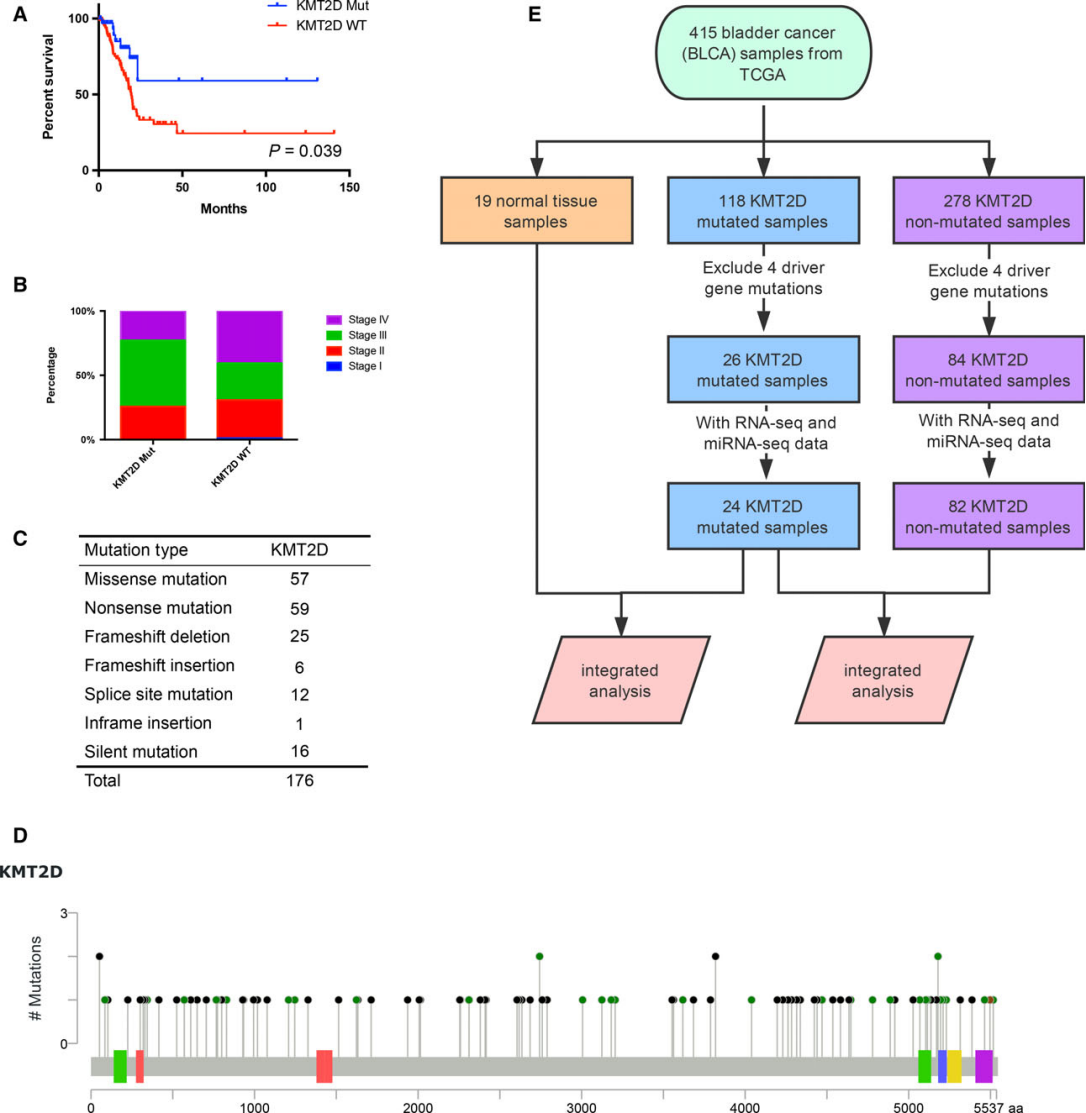
KMT2D mutations in bladder cancer. (A) Kaplan–Meier plots of overall survival associated with *KMT2D* mutation samples and *KMT2D* wild‐type samples. (B) Percentage of clinical stage of *KMT2D* mutation *vs KMT2D* wild‐type samples. (C) Frequency of each *KMT2D* mutation type. (D) The positions and domains of each *KMT2D* mutation. (E) Sample filtering workflow used for integrative genomic analysis of *KMT2D* samples.

The survival analysis between *KMT2D* mutation and *KMT2D* wild‐type samples indicated that *KMT2D* mutation was significantly related to better survival (Fig. 2A), and the proportion of stage IV in *KMT2D* mutation samples was much lower (Fig. 2B). We further conducted a genomic consequent study of *KMT2D* mutation. Four hundred and fifteen bladder cancer (BLCA) sample profiles containing mutation information were downloaded from TCGA database. One hundred and seventy‐six *KMT2D* mutations were found in 118 samples. There were 57 missense mutations, 59 nonsense mutations, 25 frameshift deletions, six frameshift insertions, 12 splice site mutations, one inframe insertion, and 16 silent mutations among them. Forty‐one tumor samples had more than one mutation, and one of these samples (TCGA‐XF‐A9T8‐01) contained five mutations (Fig. 2C,D).

As is shown in Fig. 2E, we separated all these samples into three parts including 118 *KMT2D* mutation samples, 278 *KMT2D* non‐mutation samples and 19 normal tissue samples. Next, we excluded samples with four candidate BCa driver genes (*TP53*,* PIK3CA*,* FGFR3*, and *RB1*) from downstream analysis, and this filtration procedure resulted in 26 *KMT2D* mutation samples and 84 ‘pan‐negative’ samples. We further identified the samples with matched RNA‐seq and miRNA‐seq data and obtained 24 *KMT2D* mutation samples and 82 ‘pan‐negative’ samples. Specifically, all these 24 mutation samples carried ‘loss‐of‐function’ mutation, including 10 missense mutations, nine nonsense mutations, one splice site mutation and four frame shift deletions. These samples were used to analyze the downstream effect of *KMT4D* mutation in BCa.

### Identification of *KMT2D* mutation‐associated biological processes by GSEA

**Figure 3:**
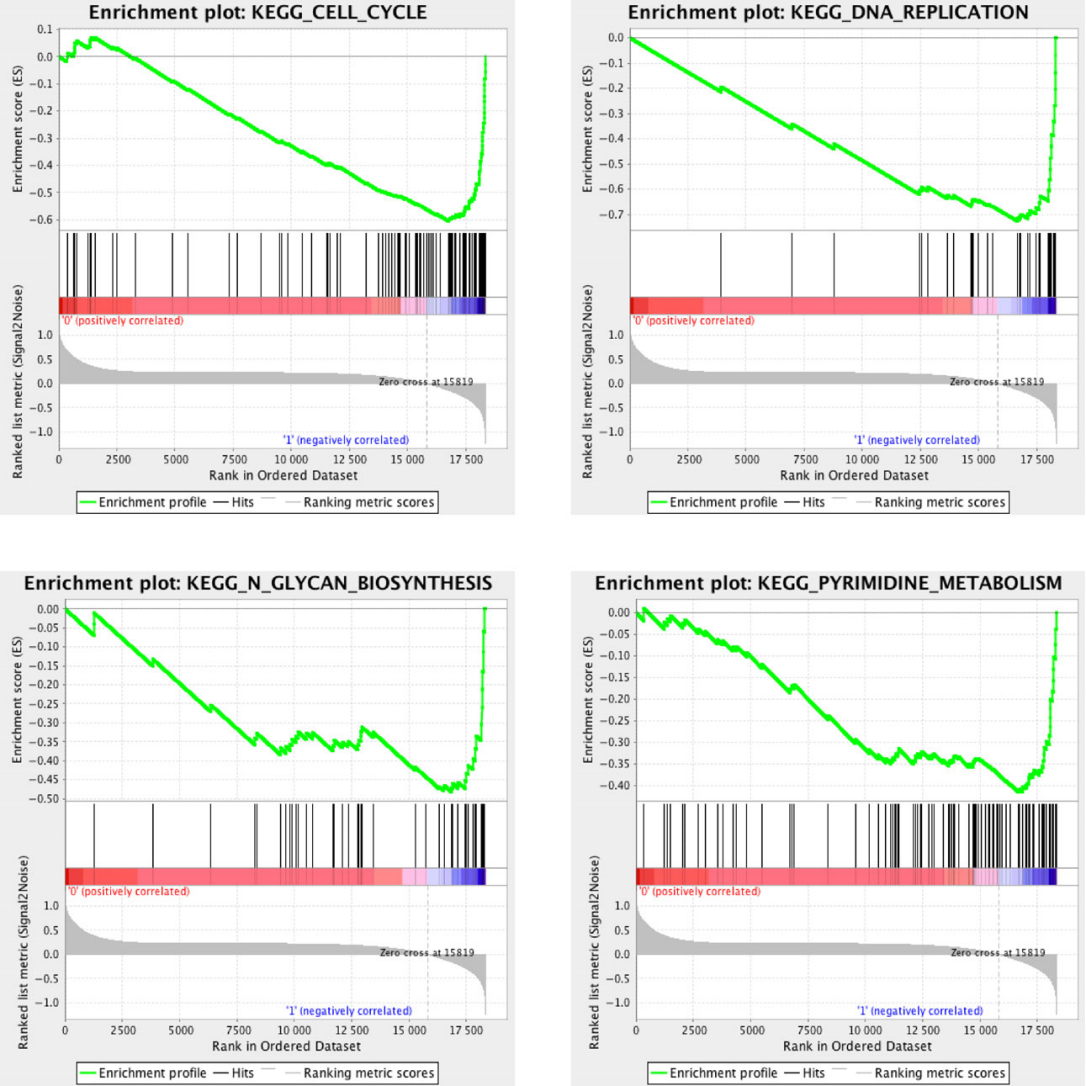
GSEA analysis of *KMT2D* mutation samples and peri‐tumor tissue samples. GSEA showed genes associated with cell cycle, DNA replication, pyrimidine metabolism and *N*‐glycan biosynthesis were significantly enriched in *KMT2D* mutation tumor samples *vs* peri‐tumor tissue samples.

To identify *KMT2D* mutation‐associated biological processes and *KMT2D* loss of function on a generalized level, GSEA was performed by comparing high throughput RNA‐sequencing TCGA cohort data of 24 *KMT2D* mutation samples and 19 normal tissue samples. Among all the predefined hallmark gene sets, cell cycle, DNA replication, pyrimidine metabolism, and *N*‐glycan biosynthesis were found to be significantly associated with *KMT2D* mutation in the TCGA cohort (Fig. 3).

### Differentially expressed genes and transcriptional factors associated with *KMT2D* mutation

**Figure 4:**
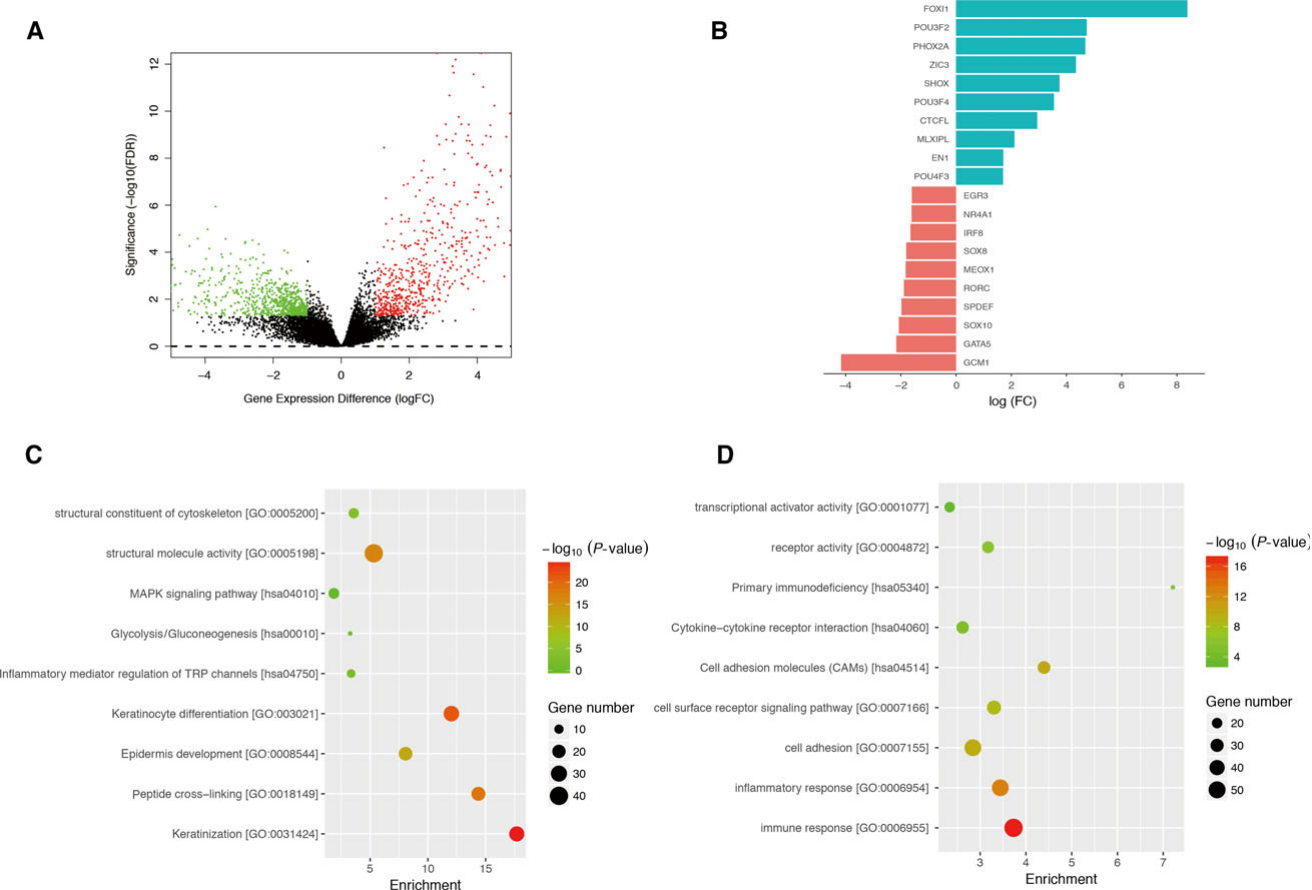
Differentially expressed genes and transcriptional factors associated with *KMT2D* mutation. (A) Volcano plot of significance of gene expression difference between *KMT2D* mutation group and ‘pan‐negative’ group at gene expression levels. Each dot represents one gene. A gene is called significantly and differentially expressed if its |log(FC)| > 1 and *P*‐value < 0.05. (B) Bar plot of log2 transformation of fold change in differentially expressed transcriptional factors (TFs). Only TFs with top 20 |log_2_(FC)| are shown. (C) Functional enrichment results of up‐regulated genes in *KMT2D* mutation group compared with ‘pan‐negative’ group. (D) Functional enrichment results of down‐regulated genes in *KMT2D* mutation group compared with ‘pan‐negative’ group.

We identified DEGs by comparing gene expression profiles of *KMT2D* mutation and pan‐negative groups using edger; 1379 DEGs in total (648 up‐regulated and 731 down‐regulated) were identified in *KMT2D* mutation samples compared with the ‘pan‐negative’ group by cutoffs: absolute value of fold change (log2 transformed) (|log_2_(FC)|) > 1 and *P* < 0.05 (Fig. 4A). Among these DEGs, 33 TFs were identified, 15 were high expressed and 18 were low expressed (Fig. 4B, Table S2). Gene function enrichment analysis indicated up‐regulated genes were significantly enriched in keratinization (*P* = 1.18E‐25), keratinocyte differentiation (*P* = 4.26E‐22), peptide cross‐linking (*P* = 2.91E‐19) and epidermis development (*P* = 7.73E‐13), while down‐regulated genes were enriched in immune response (*P* = 4.75E‐18), inflammatory response (*P* = 1.29E‐13), cell adhesion (*P* = 1.34E‐10) and cell surface receptor signaling (*P* = 3.44E‐09) (Fig. 4C,D).

### miRNA dysregulation associated with *KMT2D* mutations

**Figure 5:**
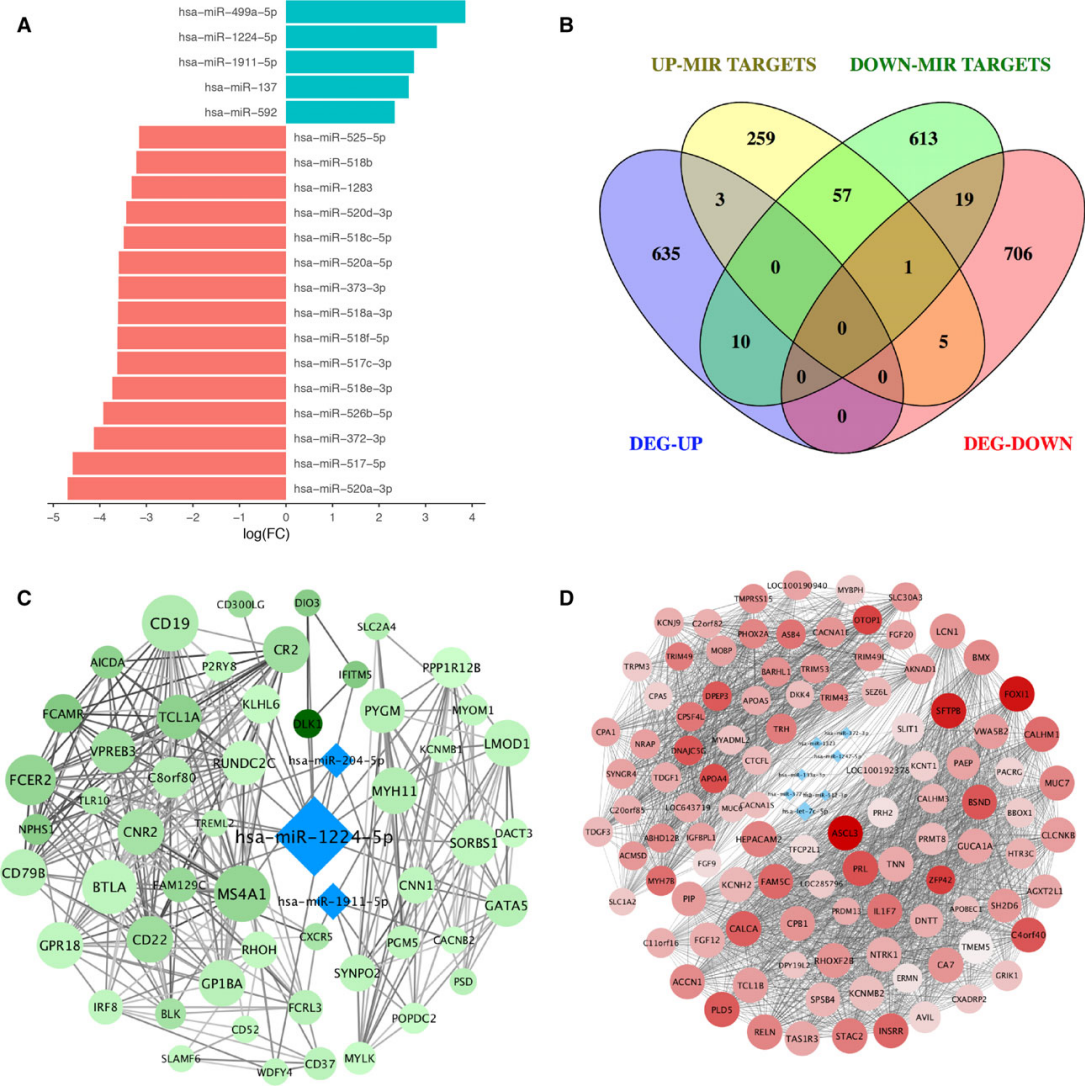
miRNA dysregulation and co‐expression networks of *KMT2D* mutation. (A) Bar plot of top 20 up‐regulated miRNAs and down‐regulated miRNAs in *KMT2D* mutation samples compared with ‘pan‐negative’ samples. (B) Venn diagram representation of the overlaps among up‐regulated genes (DEG‐up), down‐regulated genes (DEG‐down), target genes of up‐regulated miRNAs (Up miRNA targets) and target genes of down‐regulated miRNAs (Down miRNA targets). (C) *KMT2D* mutation‐specific down‐regulated co‐expression network with up‐expressed miRNAs. (D) *KMT2D* mutation‐specific up‐regulated co‐expression network with down‐expressed miRNAs. The color intensity of dot is correlated with |log_2_(FC)| of each gene and the size of dot is in proportion to the degree of dots.

Sixty‐one different expression miRNAs were found to be related to KMT2D truncation mutations based on the threshold: *P* < 0.05 and |log_2_(FC)| > 1. Thirteen miRNAs were up‐regulated in samples with *KMT2D* mutation while the other 48 miRNAs showed a down‐regulation pattern (Table S3), and the top 20 differently expressed miRNAs are illustrated in Fig. 5A. Among all the differentially expressed miRNAs, only 13 up‐regulated miRNAs and 48 down‐regulated miRNAs had corresponding targets according to databases (TarBase or miRTarBase). By overlapping miRNA targets and DEGs, we uncovered 13 up‐regulated target genes and 25 down‐regulated target genes (Fig. 5B).

### Comprehensive analysis for *KMT2D* mutations in bladder cancer

**Figure 6:**
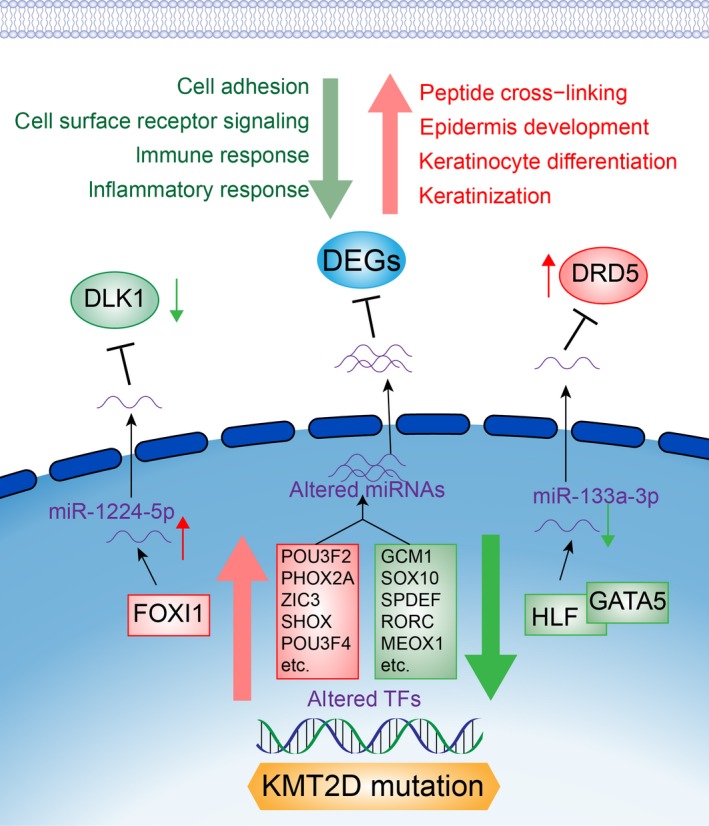
Hypothesized mechanisms of KMT2D mutation functions in the progression of bladder cancer. Up‐regulated genes and pathways are shown in red and down‐regulated genes and pathways are shown in green.

Co‐expression networks were built by r software based on mRNA expression profiles to discover potential regulatory pathways of *KMT2D* mutation‐related genes and miRNAs in BCa (Fig. 5C,D; more details is in Materials and methods). The miRNAs of those target genes that were identified as co‐expressed with other genes were brought into the network, and so were their corresponding target genes. Seven highly expressed miRNAs (miR‐1224‐5p, miR‐1911‐5p, miR‐204‐5p, miR‐203a‐3p, miR‐375, miR‐508‐3p, and miR‐592) and 39 low‐expression miRNAs (miR‐372‐3p, miR‐1323, miR‐1247‐5p, miR133a‐3p, miR‐377‐5p, miR‐512‐3p, miR‐let‐7c‐5p, etc.) were observed in down‐regulated and up‐regulated gene co‐expression networks, respectively. In the down‐regulated co‐expression networks, we noticed that miR‐1224‐5p has the highest degree level at 37, which indicated its significance in the network, and one of its possible targets was the top DEG, *DLK1* (log_2_(FC) = −12.3). An up‐regulated TF, *FOXI1*, had strong correlation with miR‐1224‐5p (cor = 0.89), enabling us to figure out a possible regulation pathway linked to *KMT2D* mutation in BCa: FOXI1–miR‐1224‐5p–DLK1. Similarly, we proposed HLF/GATA5–miR‐133a‐5p–DRD5 regulation in the up‐regulated genes co‐expression network based on high correlation coefficients and miRNA target prediction (Fig. 6).

### KMT2D knockdown inhibits viability, migration and invasion of bladder cancer cells *in vitro*

**Figure 7:**
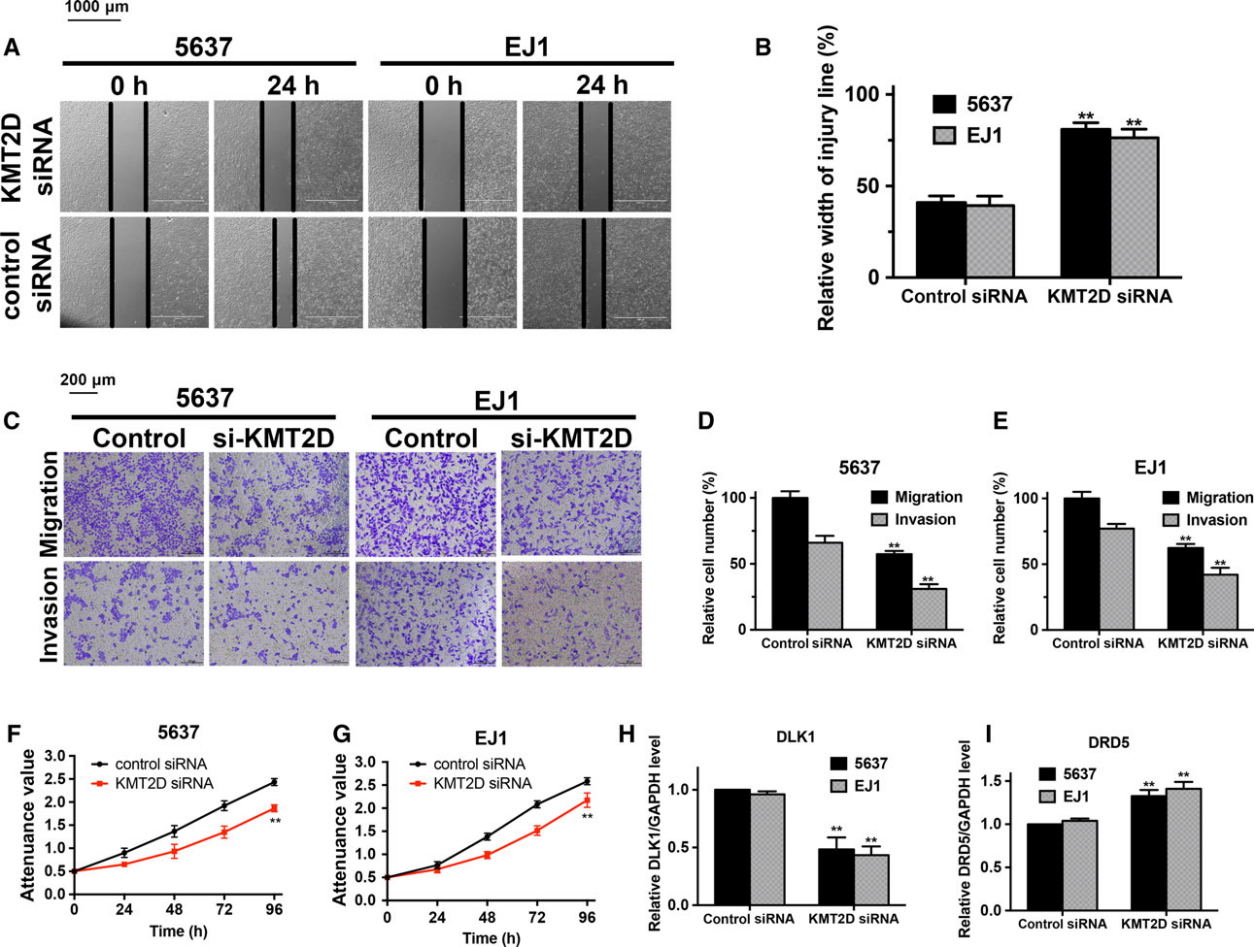
KMT2D knockdown attenuates the viability and migration and regulates the mRNA expression levels of DLDK1 and DRD5 in bladder cancer cells. (A,B) KMT2D siRNA and control siRNA transfected bladder cancer cells (5637 and EJ1 cells) were wounded by scraping. Based on the width of the wound at 0 h, the relative width at 24 h was calculated. Bar = 1000 μm. (C) 5637 and EJ1 cells transfected with KMT2D siRNA or control siRNA were subjected to migration and invasion assay. Representative photographs were taken. Bar = 200 μm. (D,E) The number of migrated and invaded cells was quantified; **significant differences, *P* < 0.01. (F,G) MTS assay revealed the growth curves of indicated cells at different time intervals. (H,I) mRNA relative expression levels of DLDK1 and DRD5 in two KMT2D knockdown cell lines (5637 and EJ1 cells) were measured by qRT‐PCR. The control cell gene expression level was set as ‘1’, and relative expression levels are shown as fold changes compared. Student's *t*‐test was performed to compare difference between groups and the results are described as mean ± standard deviation (SD) of three independent experiments. Two‐sided *P* < 0.05 was regarded as statistically significant.

KMT2D knockdown was performed in two BCa cell lines, 5637 and EJ1 cells. The wound healing, transwell migration and invasion assays were applied and revealed that KMT2D siRNA markedly inhibited the migration and invasion in 5637 and EJ1 cells (*P* < 0.05) (Fig. 7A–E). The cell viability was measured by MTS assay. As shown in Fig. 7F,G, the proliferation rate ratio of both cell lines transfected with KMT2D siRNA was dramatically reduced in comparison with cells transfected with control siRNA (*P* < 0.05).

### KMT2D knockdown negatively regulated DLK1 gene expression and up‐regulated the expression of DRD5

We examined the expressions of DLK1 and DRD5 mRNA by quantitative RT‐PCR after transfection of KMT2D siRNA or corresponding control into 5637 and EJ1 cells. The results indicated DLK1 expression level was effectively down‐regulated and DRD5 was significantly up‐regulated in KMT2D knockdown cells, which coincided with the networks we constructed (Fig. 7H,I).

## Discussion

Histone lysine methyltransferases drive different mechanisms involved in tumorigenesis and progression, including replication stress, impaired DNA repair, proliferation of tumor cells, angiogenesis, and metastasis 21, 22. The significance of genetic alteration of HMTs was reported in various kinds of tumors, including renal cancer 23, 24, lung cancer 25, 26, cervical cancer 27, and lymphoma 28. However, investigation into HMT alterations in BCa is extremely limited. In our study, we identified six HMTs (*ASH1L*,* PRDM9*,* SETD3*,* SETD5*,* WHSC1L1*, and *KMT2D*) that might play a critical role in oncogenesis and prognosis in BCa. In contrast to surgical treatment, the possibility of reversing epigenetic changes provides the initial goal for therapeutic approaches to BCa.

In BCa patients, *WHSC1L1* exhibited both high‐level amplification alteration and heterozygous deletion alteration when the correlation coefficient of mRNA expression and CNA was over 0.5, indicating genetic alteration of *WHSC1L1* is unstable in BCa and consistent with its expression level. Intriguingly, Kang *et al*. 29 found that *WHSC1L1* was up‐regulated in various human cancers including bladder carcinoma, which was not consist with our findings that *WHSC1L1* was slightly elevated in BCa. The possible reason is that we relied on the TCGA database for which the patients came from North America while Kang's research enrolled Japanese patients; the geographical differences may lead to the inconsistence in *WHSC1L1* expression level.

In our study and other researches, *KMT2D* had the highest frequency of mutation among HMTs. Nearly 28.4% patients had somatic mutation indicating that *KMT2D* mutation might be an individual factor predicting the occurrence and prognosis of BCa. Therefore, we performed a Kaplan–Meier analysis and the results indicated that the *KMT2D* mutation group had significantly longer survival and better prognosis than the *KMT2D* non‐mutation group. Moreover, Froimchuk *et al*. found that *KMT2D* plays critical roles in tumor development and it is frequently mutated in various forms of cancer, playing harmful roles in tumor progression 30. That *KMT2D* mutation patients had longer survival in BCa is a crucial finding, and thus we compared the mRNA expression profiles of mutation and non‐mutation groups. Surprisingly, we uncovered two signaling pathways leading to tumor suppression in the mutation group, FOX1–miR‐1224‐5p–DLK1 and HIF/GATA5–miR‐133a‐3p–DRD5. *DLK1*, the tumor activator, was down‐regulated in the *KMT2D* mutation group while *DRD5* was up‐regulated and acted in the opposite way; the expression change trends were validated by qRT‐PCR results. That DLK1 promotes lung cancer cell invasion through up‐regulation of matrix metalloprotease 9 expression, and that up‐regulation of *DRD5* inhibits tumor growth by autophagic cell death have been reported in recent year 31, 32. These findings may explain why the *KMT2D* mutation group had a better prognosis.

Epigenetic regulation and genetic changes plays an important role in the occurrence and development of cancer 33. Contrary to genetic disorders, the possibility of reversing epigenetic changes provides the initial goal for therapeutic applications. Over the past few years, efforts have been devoted to the balance of epigenetic regulation using epi‐drugs to produce new strategies for cancer therapy, hoping to restore pharmacological effects 34. Histone acetylation and DNA methylation are epigenetic modifications that are closely related to the pathology of human cancer, and development of the two kinds of enzyme inhibitors is in hand. Recent discoveries in chemistry and biomedical applications have led to new targeted therapies and histone methylation is a promising therapeutic target for BCa therapy 12.

## Conflict of interest

The authors declare no conflict of interest.

## Author contributions

Conception and design: BD, LY, DX; financial support: HX, ZY; administrative support: DX, HX, ZY; data analysis and interpretation: BD, YZ; manuscript writing: BD, LY, ZW; final approval of manuscript: all authors.

## Supporting information


**Fig. S1.** The expression profiles of 50 HMTs in BCa tumor and peri‐tumor tissues.

**Table S1.** Frequency of HMT copy number alterations and mutations (%).

**Table S2.** Differentially expressed transcription factors between *KMT2D* mutation group and control group.

**Table S3.** Differentially regulated microRNAs between *KMT2D* mutation group and control group.
