# Supplementary material for: Analysis of the role of mutations in the KMT2D histone lysine methyltransferase in bladder cancer
Source: FEBS Open Bio. 2019 Feb 21;9(4):693–706. doi: 10.1002/2211-5463.12600 (PMC6443872; doi:10.1002/2211-5463.12600)
Supplement: Supplementary file 1 — Fig. S1. The expression profiles of 50 HMTs in BCa tumor and peri‐tumor tissues. [file FEB4-9-693-s001.pdf]

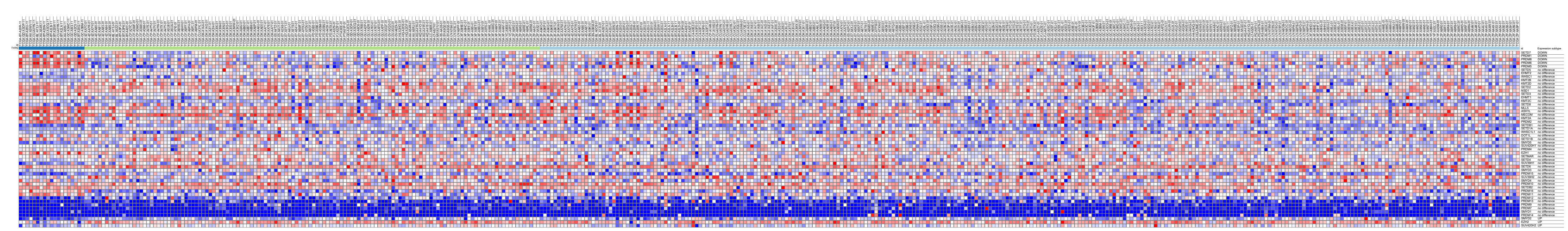

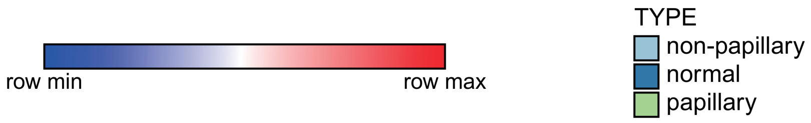

Supplementary Figure.1
The expression profiles of 50 HMTs in BLCA tumor and peri-tumor tissues.
