# Supplementary material for: Analysis of the role of mutations in the KMT2D histone lysine methyltransferase in bladder cancer
Source: FEBS Open Bio. 2019 Feb 21;9(4):693–706. doi: 10.1002/2211-5463.12600 (PMC6443872; doi:10.1002/2211-5463.12600)
Supplement: Supplementary file 2 — Table S1. Frequency of HMT copy number alterations and mutations (%). [file FEB4-9-693-s002.docx]

Supplementary Table.1: Frequency of HMT copy number alterations and mutations (%)

| Gene | Amp | Gain | Diploid | Hetloss | Homdel | Mutation |
| --- | --- | --- | --- | --- | --- | --- |
| SETDB1 | 10.78 | 38.48 | 46.57 | 4.17 | 0.00 | 3.08 |
| WHSC1L1 | 9.56 | 21.32 | 33.09 | 33.82 | 2.21 | 1.54 |
| SETD5 | 9.31 | 33.58 | 43.38 | 13.73 | 0.00 | 2.31 |
| PRDM9 | 7.60 | 41.42 | 41.67 | 9.07 | 0.25 | 5.38 |
| ASH1L | 5.64 | 39.46 | 49.02 | 5.64 | 0.25 | 7.69 |
| SETMAR | 5.39 | 33.58 | 44.85 | 16.18 | 0.00 | 0.00 |
| MECOM | 4.90 | 47.79 | 42.65 | 3.92 | 0.74 | 5.38 |
| PRDM14 | 4.41 | 50.25 | 38.48 | 6.86 | 0.00 | 2.31 |
| SUV420H1 | 3.92 | 23.04 | 53.92 | 18.87 | 0.25 | 5.38 |
| SUV39H2 | 3.68 | 33.58 | 49.75 | 11.76 | 1.23 | 0.00 |
| SUV420H2 | 3.68 | 35.54 | 46.81 | 13.97 | 0.00 | 0.00 |
| KMT2B | 3.43 | 36.03 | 48.04 | 12.01 | 0.49 | 9.23 |
| WHSC1 | 2.70 | 14.22 | 50.00 | 32.35 | 0.74 | 5.38 |
| PRDM7 | 1.96 | 20.10 | 50.00 | 26.96 | 0.98 | 2.31 |
| EHMT2 | 1.96 | 25.00 | 53.92 | 18.63 | 0.49 | 3.08 |
| SMYD5 | 1.96 | 30.88 | 59.07 | 8.09 | 0.00 | 0.00 |
| SMYD3 | 1.72 | 32.11 | 52.94 | 12.25 | 0.98 | 0.77 |
| PRDM1 | 1.47 | 6.13 | 47.55 | 43.14 | 1.72 | 1.54 |
| SETD4 | 1.47 | 29.90 | 49.26 | 19.12 | 0.25 | 1.54 |
| PRDM11 | 1.23 | 8.09 | 48.77 | 41.42 | 0.49 | 1.54 |
| PRDM8 | 1.23 | 8.58 | 56.13 | 34.07 | 0.00 | 0.00 |
| SETD1A | 1.23 | 20.59 | 54.90 | 23.28 | 0.00 | 2.31 |
| SETD1B | 1.23 | 23.28 | 58.33 | 17.16 | 0.00 | 0.00 |
| SETD2 | 0.98 | 25.49 | 47.55 | 25.00 | 0.98 | 6.92 |
| PRDM2 | 0.98 | 19.12 | 61.27 | 18.63 | 0.00 | 5.38 |
| PRDM4 | 0.98 | 22.30 | 59.07 | 17.65 | 0.00 | 2.31 |
| SETD8 | 0.98 | 22.55 | 59.31 | 17.16 | 0.00 | 1.54 |
| SMYD1 | 0.98 | 28.92 | 61.27 | 8.82 | 0.00 | 1.54 |
| SMYD4 | 0.74 | 11.27 | 38.24 | 47.79 | 1.96 | 3.85 |
| KMT2C | 0.74 | 30.88 | 54.66 | 12.01 | 1.72 | 20.77 |
| EHMT1 | 0.74 | 15.44 | 44.12 | 38.97 | 0.74 | 1.54 |
| SETD7 | 0.74 | 7.84 | 51.72 | 39.22 | 0.49 | 0.00 |
| PRDM5 | 0.74 | 7.35 | 53.68 | 37.99 | 0.25 | 3.08 |
| DOT1L | 0.74 | 13.24 | 50.98 | 34.80 | 0.25 | 5.38 |
| PRDM16 | 0.74 | 21.32 | 59.07 | 18.63 | 0.25 | 6.15 |
| KMT2E | 0.74 | 34.80 | 54.66 | 9.56 | 0.25 | 5.38 |
| EZH1 | 0.74 | 36.03 | 55.64 | 7.60 | 0.00 | 1.54 |
| SETDB2 | 0.49 | 15.20 | 46.57 | 30.64 | 7.11 | 0.77 |
| SETD3 | 0.49 | 17.89 | 52.21 | 27.70 | 1.72 | 1.54 |
| NSD1 | 0.49 | 6.37 | 44.12 | 47.55 | 1.47 | 5.38 |
| PRDM10 | 0.49 | 11.52 | 51.47 | 35.78 | 0.74 | 1.54 |
| KMT2A | 0.49 | 10.78 | 51.23 | 37.01 | 0.49 | 7.52 |
| PRDM15 | 0.49 | 29.90 | 47.79 | 21.32 | 0.49 | 3.08 |
| SUV39H1 | 0.49 | 16.67 | 62.50 | 19.85 | 0.49 | 0.00 |
| EZH2 | 0.49 | 31.62 | 54.90 | 12.50 | 0.49 | 2.31 |
| KMT2D | 0.49 | 20.34 | 62.01 | 16.91 | 0.25 | 16.90 |
| PRDM6 | 0.25 | 6.86 | 45.34 | 46.08 | 1.47 | 0.00 |
| PRDM13 | 0.25 | 6.13 | 48.77 | 43.38 | 1.47 | 0.00 |
| PRDM12 | 0.25 | 13.97 | 44.85 | 40.20 | 0.74 | 0.00 |
| SMYD2 | 0.25 | 31.62 | 53.19 | 14.22 | 0.74 | 0.77 |
| SETD6 | 0.25 | 18.63 | 50.49 | 30.15 | 0.49 | 0.77 |

Footnote: Amp=high-level amplification; Gain=low-level gain; Hetless=heterozygous deletion; Homdel=homozygous deletion. Genes were ranked based on the frequency of high-level amplification.
